# Miscellaneous Notes

**Published:** 1880-01

**Authors:** 


### MISCELLANEOUS NOTES.

Iridium is the most difficult metal to fuse.——Atmospheric electricity is said to exert a beneficial influence on vegetation.——The highest point reached on the railway over the Raton mountains is at Raton's Pass; an elevation of 7,720 feet above the sea-level.——"The first train was run over the Second Avenue Elevated Railway, New York, December 15th.——During the second week of December, the price of crude rubber rose from 50 cents, its old price, to $1.00 per pound.——Recent statistics show a gradual increase in the manufactures of the South.——Eye-glasses are now fitted with gum-elastic, to prevent irritation of the nose.——Upon examining the lungs of a dead frog, Dr. W. B. Fletcher found innumerable black carbon crystals, and his clever theory is that in the ante-deluvian period, huge reptiles may in the same way have furnished the material for the diamond.——Puchot has found that the iodine test for starch does not work well in the presence of some of the organic nitrogenous substances.——The French make wood durable by impregnating it with lime.——According to a writer in the "*Science Gossip*" "the cucumber is known to have been cultivated for more than three thousand years."——A new coloring matter called *ericine*, is made from poplar wood.——The annual production of alum and sulphate of alumina in France, exceeds the national wants by 4,000 tons.——M. Claude Etienne Minie, famous for his improvements in firearms, died recently in Paris.——The Inman steamship, City of Berlin, was the first to use the electric light, employing six candles and satisfactorily to the passengers.——According to many who have studied the Chinese in their own country, there is much in their civilization to commend.——There are eight hundred and thirty-five persons in the United States engaged in the study of entomology.——The mean depth of the sea is estimated at 1,877 fathoms, equal to

11,262 feet.——Herbert Spencer is again at work upon the "Principles of Sociology"——Dr. Burnett an opthalmologist in Washington, thinks the color-sense should be educated in the schools.——Figures are inadequate to express the numbers of herrings annually consumed by their enemies in the seas.——The fabled Arabian city of El-Hejjer, turns out to have been merely a cluster of several palm villages in clay.——Sir John Lubbock is an earnest advocate of science-teaching in the schools.——Dr. Edward Yenzl of Vienna an eminent botanist, died recently, aged 72 years.——Nordenskjold is going on another exploring trip to the Siberian Polar Sea.—-Spencer's "Data of Ethics" has been translated into Russian, and will be published in the successive issues of the "Svet."——Cincinnati manufactured 24,766,328 pounds of starch during the last year.-—The "Society of Pharmacy for the Ottoman Empire" was formed in June last and reports 140 members.—-The artificial propagation of sponges in Austria, has, it is said, received official recognition from the government.

